# Supplementary material for: The transcription factor ZIP-1 promotes resistance to intracellular infection in Caenorhabditis elegans
Source: Nat Commun. 2022 Jan 10;13:17. doi: 10.1038/s41467-021-27621-w (PMC8748929; doi:10.1038/s41467-021-27621-w)
Supplement: Supplementary file 1 — Supplementary Information [file 41467_2021_27621_MOESM1_ESM.pdf]

The transcription factor ZIP-1 promotes resistance to intracellular infection in *Caenorhabditis elegans*

Lažetić et al.

Supplementary information

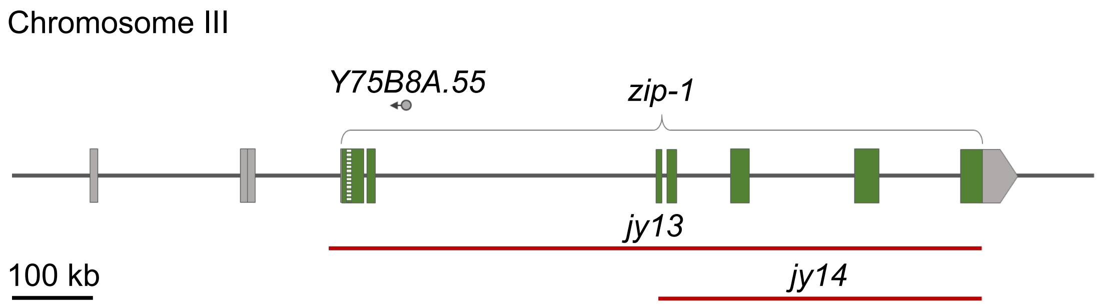

Supplementary Fig. 1: Graphic representation of the *zip-1* gene. Green boxes indicate exons; the box with green stripes represents the part of the gene that is spliced in some *zip-1* isoforms. Grey boxes represent 5’ UTR regions annotated for different *zip-1* isoforms, as well as 3’ UTR. Red lines indicate regions deleted in *jy13* and *jy14* alleles. *Y75B8A.55* non-coding RNA is indicated with a circle and an arrow.

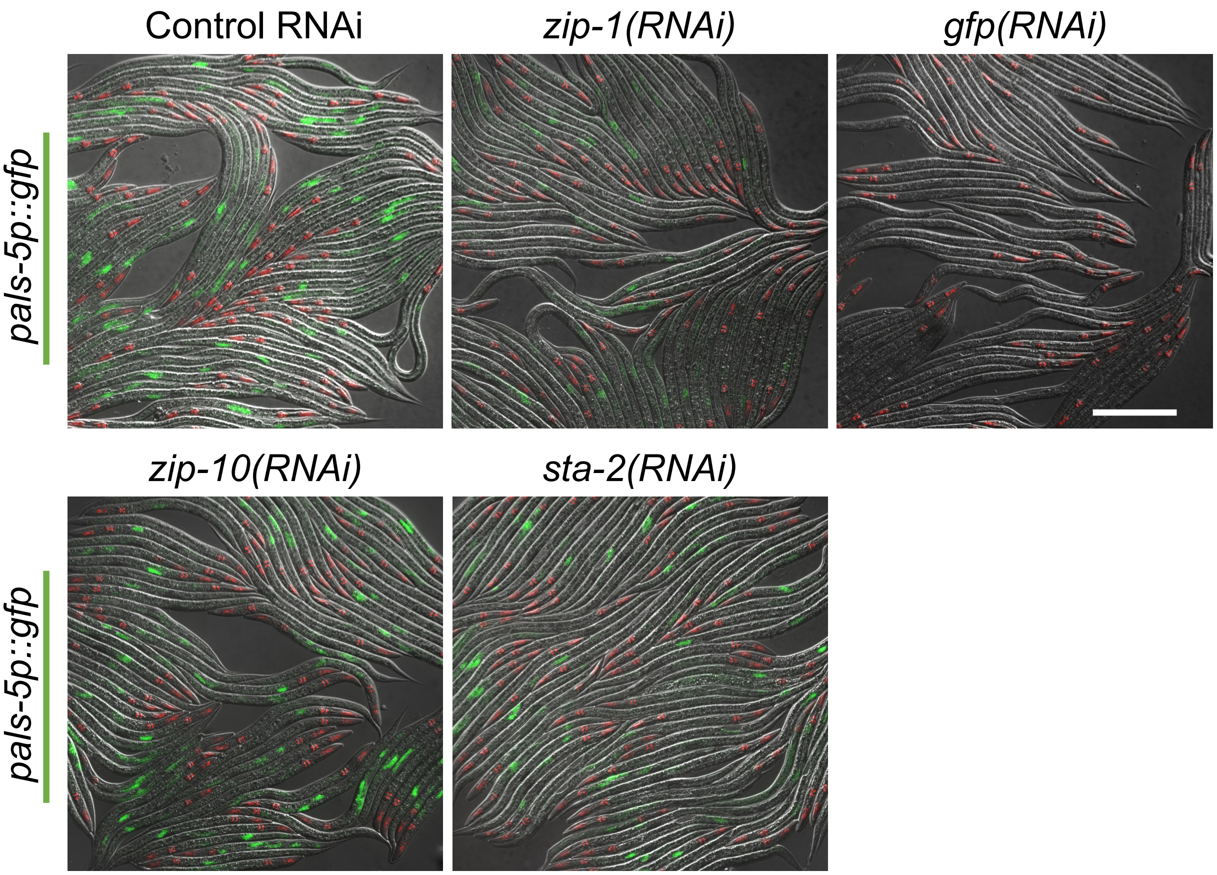

Supplementary Fig. 2: *pals-5*p::GFP expression in animals treated with *zip-10* and *sta-2* RNAi followed by prolonged heat stress. Two independent experimental replicates were performed with similar results. Fluorescent and DIC images were merged. *myo-2*p::mCherry is expressed in the pharynx and is a marker for the presence of the *jyIs8* transgene. Scale bar = 200 µm.

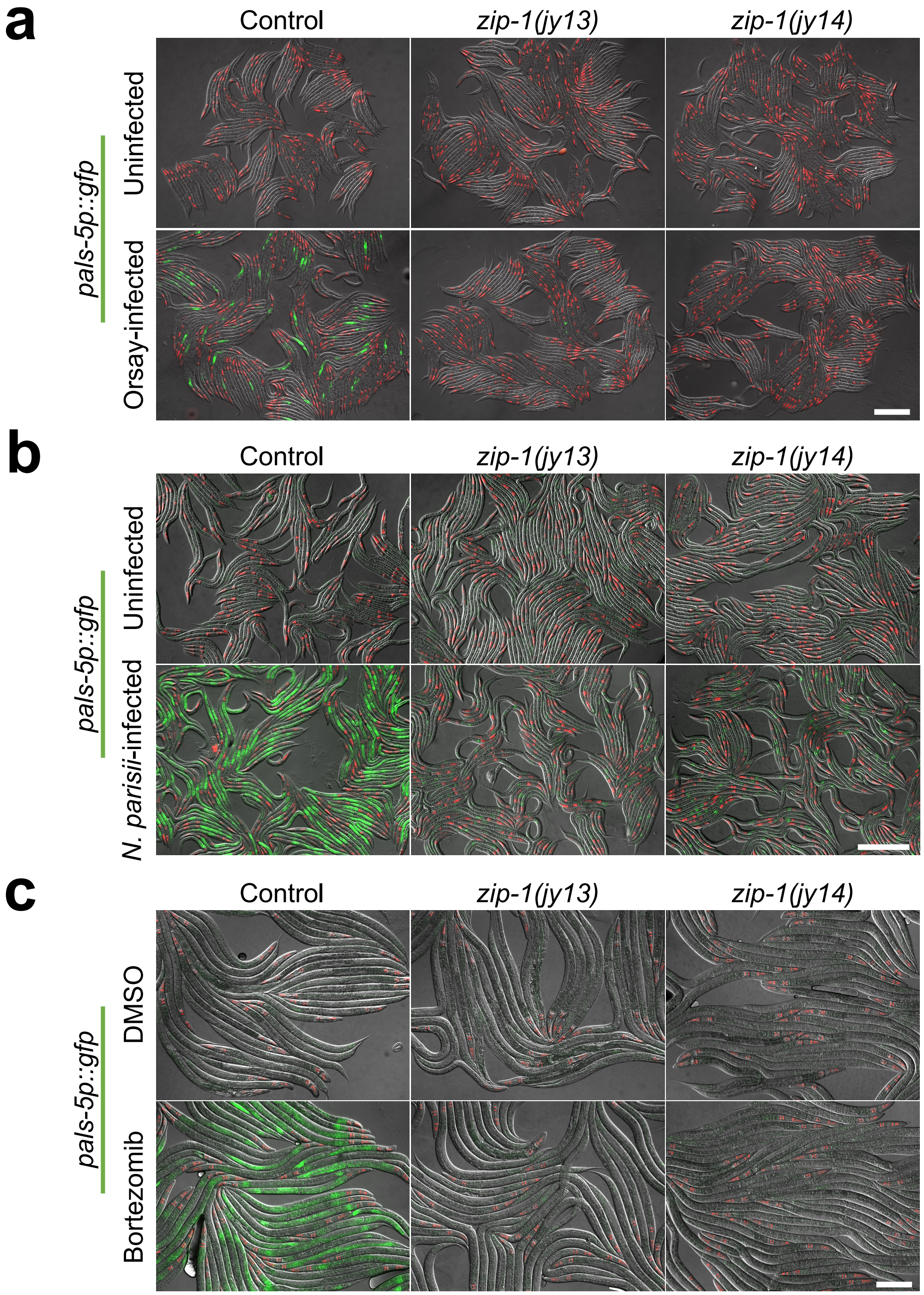

Supplementary Fig. 3: Induction of *pals-5*p::GFP expression is reduced in *zip-1(jy14)* mutants following intracellular infection with Orsay virus or *N. parisii*, as well as following proteasome inhibition by bortezomib. a-c Representative images of control (upper row) and Orsay virus-infected (**a**), *N. parisii*-infected (**b**) and bortezomib-treated animals (**c**) (lower row). Two independent experimental replicates were performed with similar results. *myo-2*p::mCherry is expressed in the pharynx and is a marker for the presence of the *jyIs8* transgene. Fluorescent and DIC images were merged. Scale bars = 200 μm.

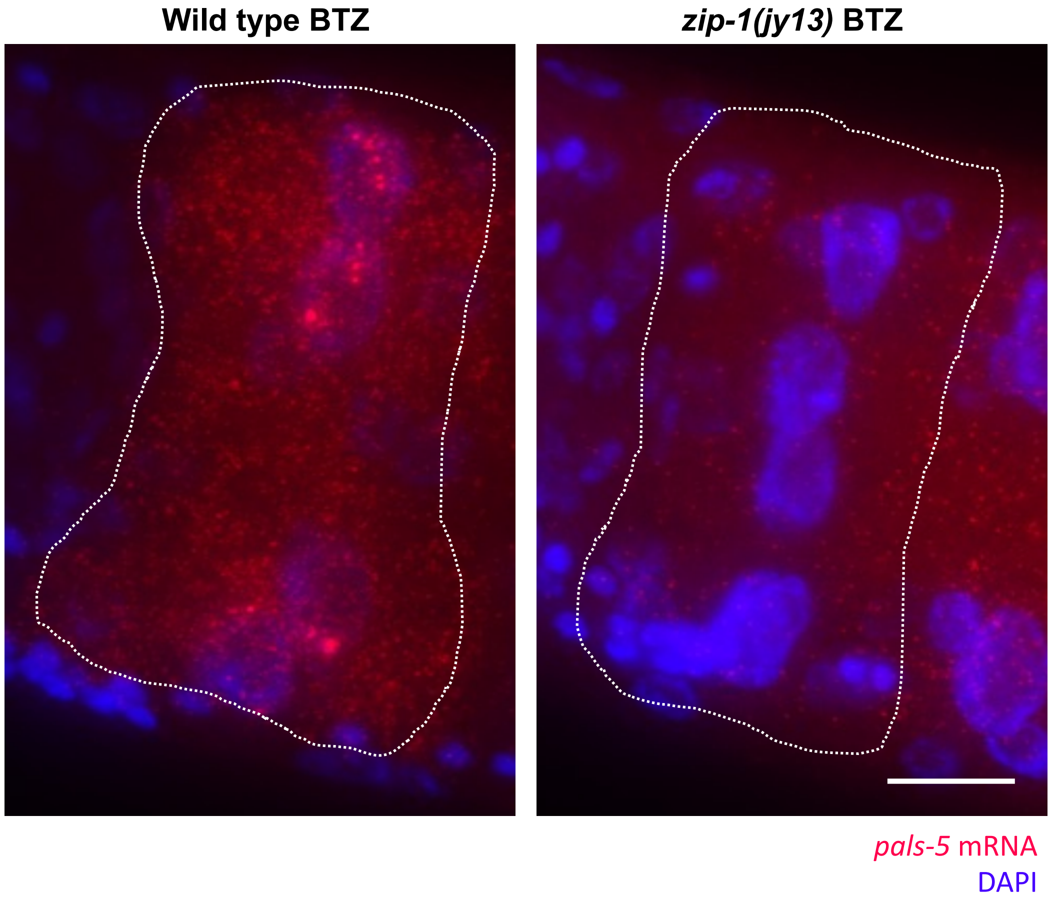

Supplementary Fig. 4: Representative images of the first four intestinal cells of bortezomib-treated animals from smFISH analysis. Three independent experimental replicates were performed with similar results. *pals-5* mRNA is visualized with a far-red fluorophore and nuclei are labeled with DAPI (blue). Images are maximal projections of z-stacks taken in far-red and blue channels. Dotted lines demarcate areas of the first four intestinal cells that were analyzed. Scale bar = 10 µm.

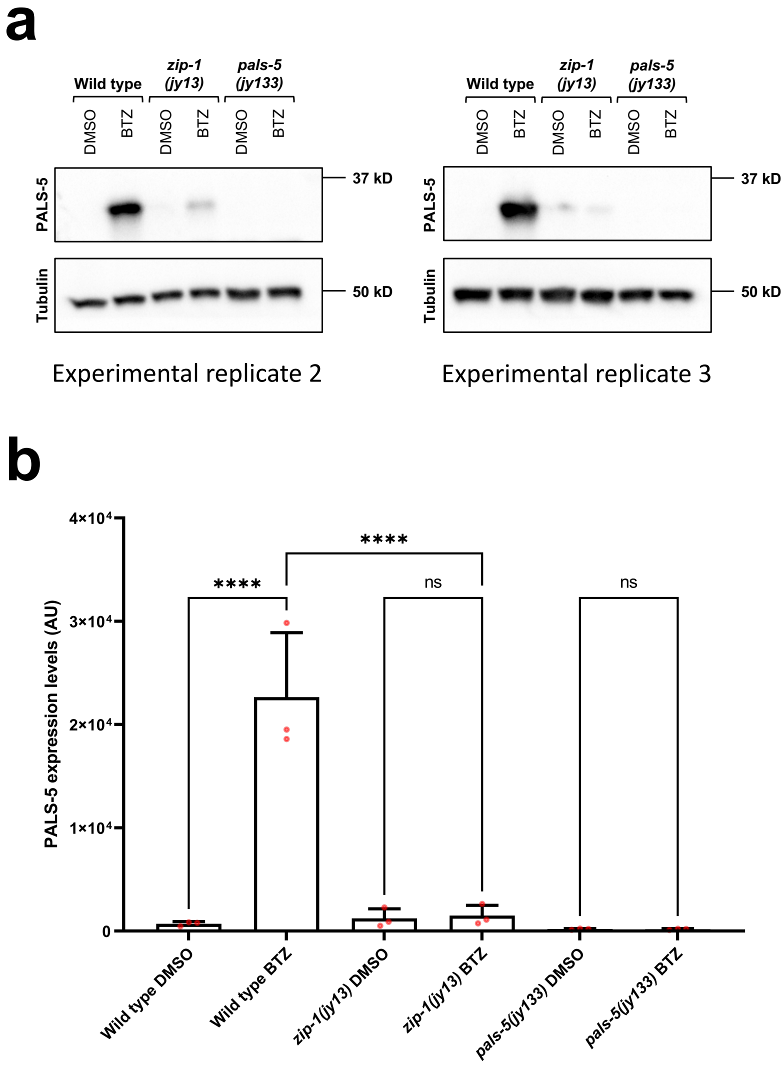

Supplementary Fig. 5: Western blot analysis of PALS-5 expression in wild-type, *zip-1(jy13)* and *pals-5(jy133)* animals. a Western blot images of the second and third experimental replicates of PALS-5 protein expression analysis in wild-type, *zip-1(jy13)* and *pals-5(jy133)* animals treated with DMSO and bortezomib. PALS-5 was detected using anti-PALS-5 antibody, whereas anti-tubulin antibody was used as a loading control. Predicted sizes are 35.4 kD for PALS-5 and around 50 kD for different members of tubulin family. Two out of three independent experimental replicates are shown; similar results were obtained from all three replicates (the remaining replicate is shown in Figure 3d). Uncropped scans of the blot are supplied in the Source Data file. **b** Quantification of PALS-5 protein expression levels in wild-type, *zip-1(jy13)* and *pals-5(jy133)* animals treated with DMSO and bortezomib. Bar height indicates mean expression value for each sample; red dots indicate values from each of three experimental replicates; error bars represent standard deviations. Strain and treatment information are indicated on x axis; PALS-5 protein levels normalized to tubulin levels are indicated on y axis in arbitrary units (AU). Statistical analysis was performed using an ordinary one-way ANOVA test to calculate *p*-values; **** $p < 0.0001$; ns indicates nonsignificant difference ($p > 0.05$). *p*-value for WT DMSO vs WT BTZ and WT BTZ vs *zip-1(jy13)* BTZ $p < 0.0001$; *zip-1(jy13)* DMSO vs *zip-1(jy13)* BTZ and *pals-5(jy133)* DMSO vs *pals-5(jy133)* BTZ $p > 0.9999$. Source data are provided as a Source Data file.

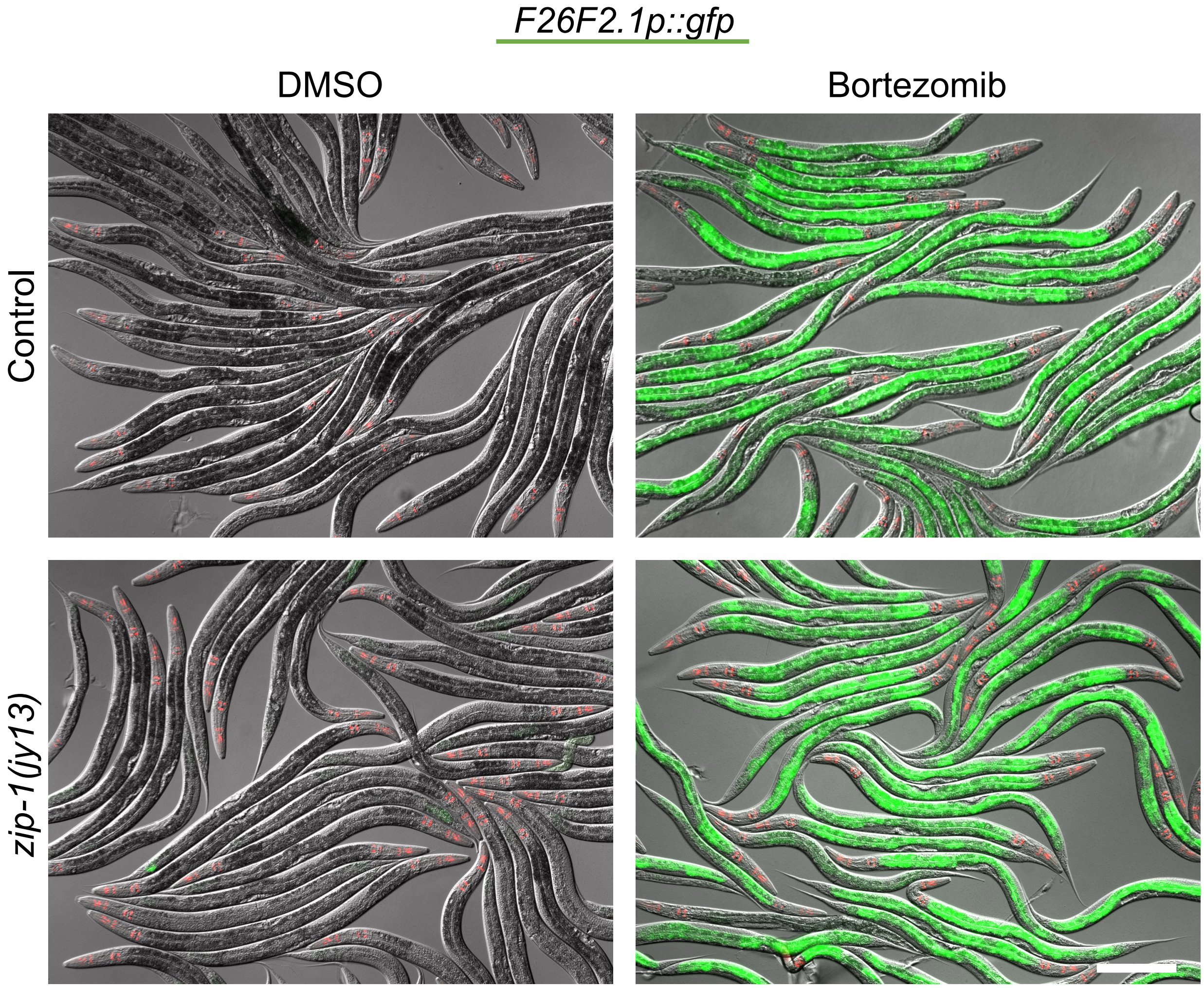

Supplementary Fig. 6: Proteasome inhibition by bortezomib induces *F26F2.1*p::GFP expression in a *zip-1(jy13)* background. Two independent experimental replicates were performed with similar results. Fluorescent and DIC images were merged. *myo-2*p::mCherry is expressed in the pharynx and is a marker for the presence of the *jyIs8* transgene. Scale bar = 200 μm.

a

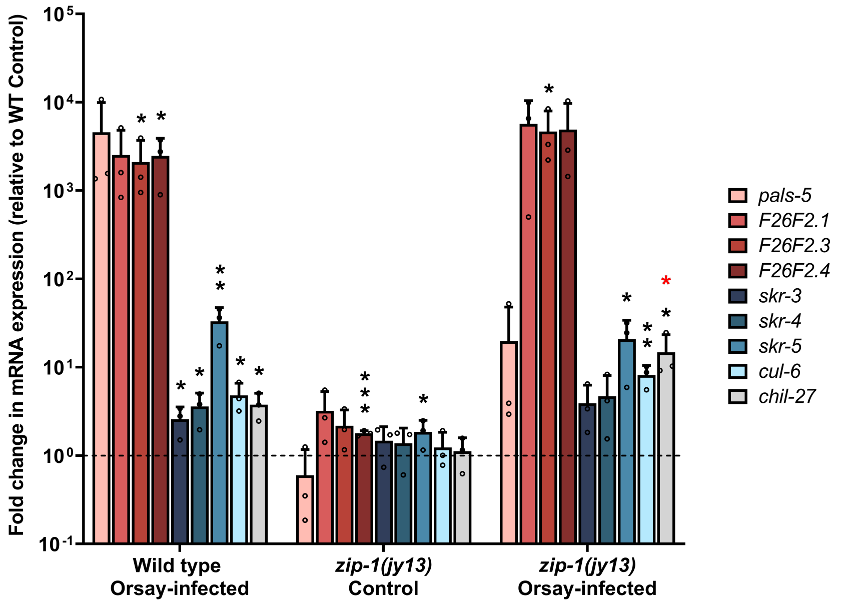

b

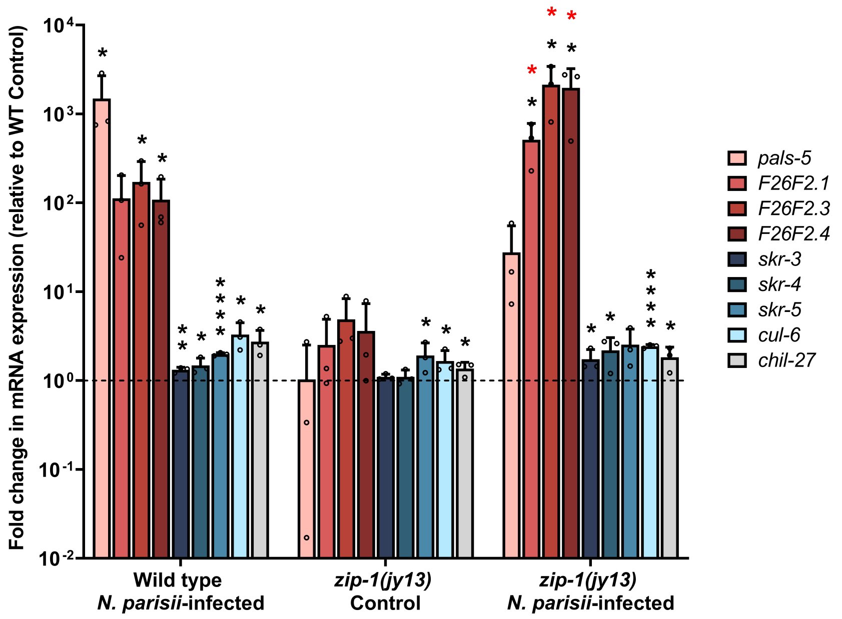

Supplementary Fig. 7: *zip-1* regulates expression of some IPR genes following intracellular infection. a, b qRT-PCR measurements of selected IPR genes and *chil-27* in wild-type and *zip-1(jy13)* animals following Orsay virus (**a**) and *N. parisii* infection (**b**). The results are shown as the fold change in gene expression relative to uninfected control strain. Three independent experimental replicates were analyzed, the values for each replicate are indicated with circles. Bar heights indicate mean values and error bars extend above. Error bars represent standard deviations. A one-tailed t-test was used to calculate p-values; black asterisks represent significant difference between the labeled sample and the uninfected wild-type control; red asterisks represent significant difference between infected *zip-1(jy13)* and infected wild-type samples; **** $p < 0.0001$; *** $p < 0.001$; ** $p < 0.01$; * $0.01 < p < 0.05$; p-values higher than 0.05 are not labeled.

p-values in **a** for WT infected vs WT control: *F26F2.3* p = 0.0429, *F26F2.4* p = 0.0206, *skr-3* p = 0.0247, *skr-4* p = 0.0211, *skr-5* p = 0.0087, *cul-6* p = 0.0111, *chil-27* p = 0.0109; *zip-1(jy13)* control vs WT control: *F26F2.4* p = 0.0002, *skr-5* p = 0.0451; *zip-1(jy13)* infected vs WT control: *F26F2.3* p = 0.0356, *skr-5* p = 0.0312, *cul-6* p = 0.0031, *chil-27* p = 0.0255; *zip-1(jy13)* infected vs WT infected: *chil-27* p = 0.0476. *p*-values in **b** for WT infected vs WT control: *pals-5* p = 0.0498, *F26F2.3* p = 0.0347, *F26F2.4* p = 0.0353, *skr-3* p = 0.0020, *skr-4* p = 0.0291, *skr-5* p < 0.0001, *cul-6* p = 0.0144, *chil-27* p = 0.0165; *zip-1(jy13)* control vs WT control: *skr-5* p = 0.0496, *cul-6* p = 0.0488, *chil-27* p = 0.0295; *zip-1(jy13)* infected vs WT control: *F26F2.1* p = 0.0154, *F26F2.3* p = 0.0234, *F26F2.4* p = 0.0279, *skr-3* p = 0.0325, *skr-4* p = 0.0371, *cul-6* p < 0.0001, *chil-27* p = 0.0308; *zip-1(jy13)* infected vs WT infected: *F26F2.1* p = 0.0363, *F26F2.3* p = 0.0299, *F26F2.4* p = 0.0327. All significant and non-significant *p*-values are provided as a Source Data file. Source data are provided as a Source Data file.

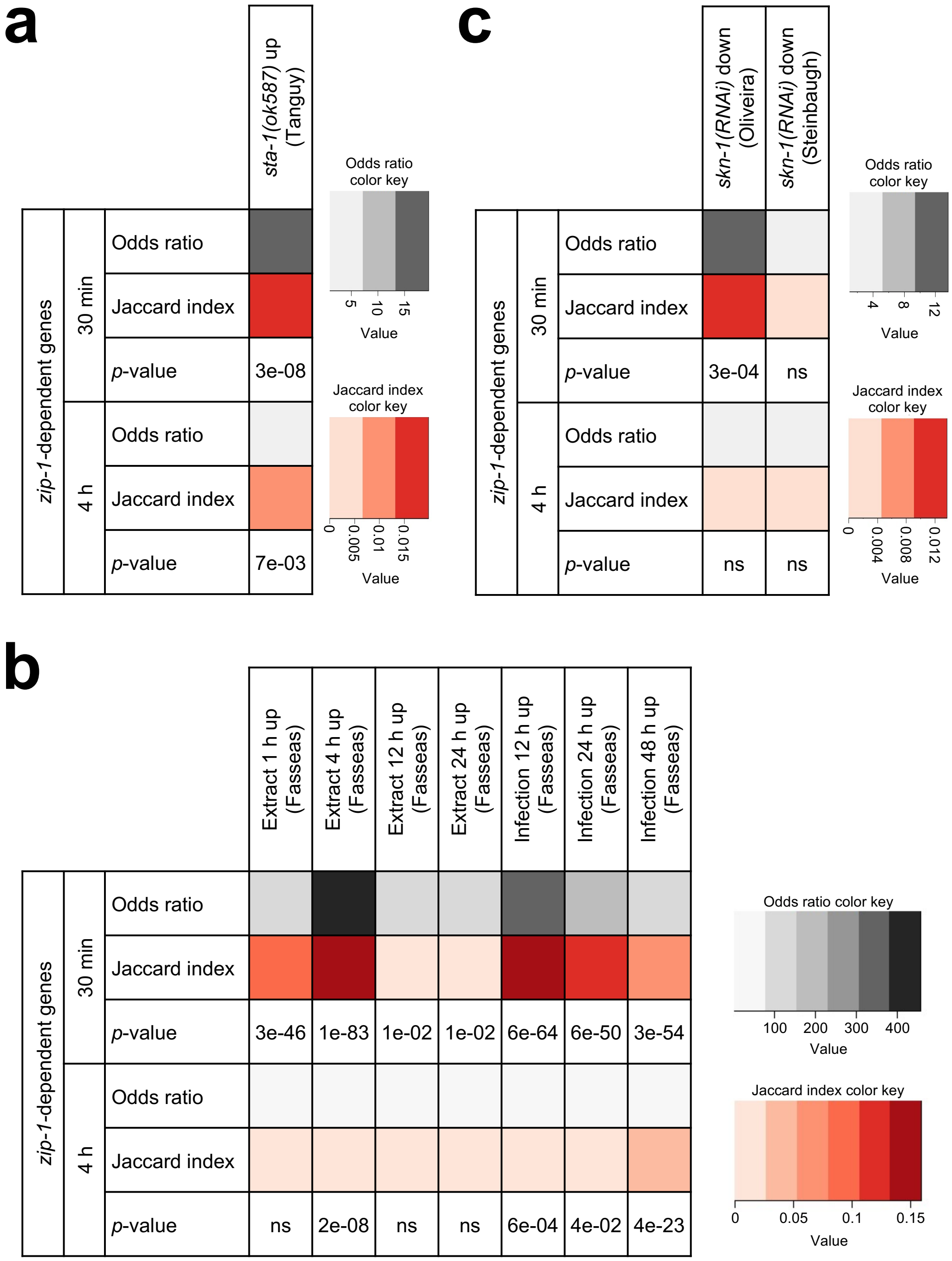

Supplementary Fig. 8: Correlation between *zip-1*-dependent genes and *sta-1*-regulated, ORR and *skn-1*-regulated genes. a-c Statistical similarity between *zip-1*-dependent gene set and genes downregulated in *sta-1(ok587)* mutants (**a**), ORR genes (**b**) and genes upregulated following *skn-1* downregulation (**c**). Fisher's exact test was used to calculate odds ratios and *p*-values. If odds ratio is greater than one, two data sets are positively corelated. Jaccard index measures similarity between two sets, with the range 0-1 (0 – no similarity, 1 – same datasets). For approximate quantification, the odds ratio and Jaccard index color keys are indicated on the right side of each table. Source data are provided as a Supplementary Data 3 file.

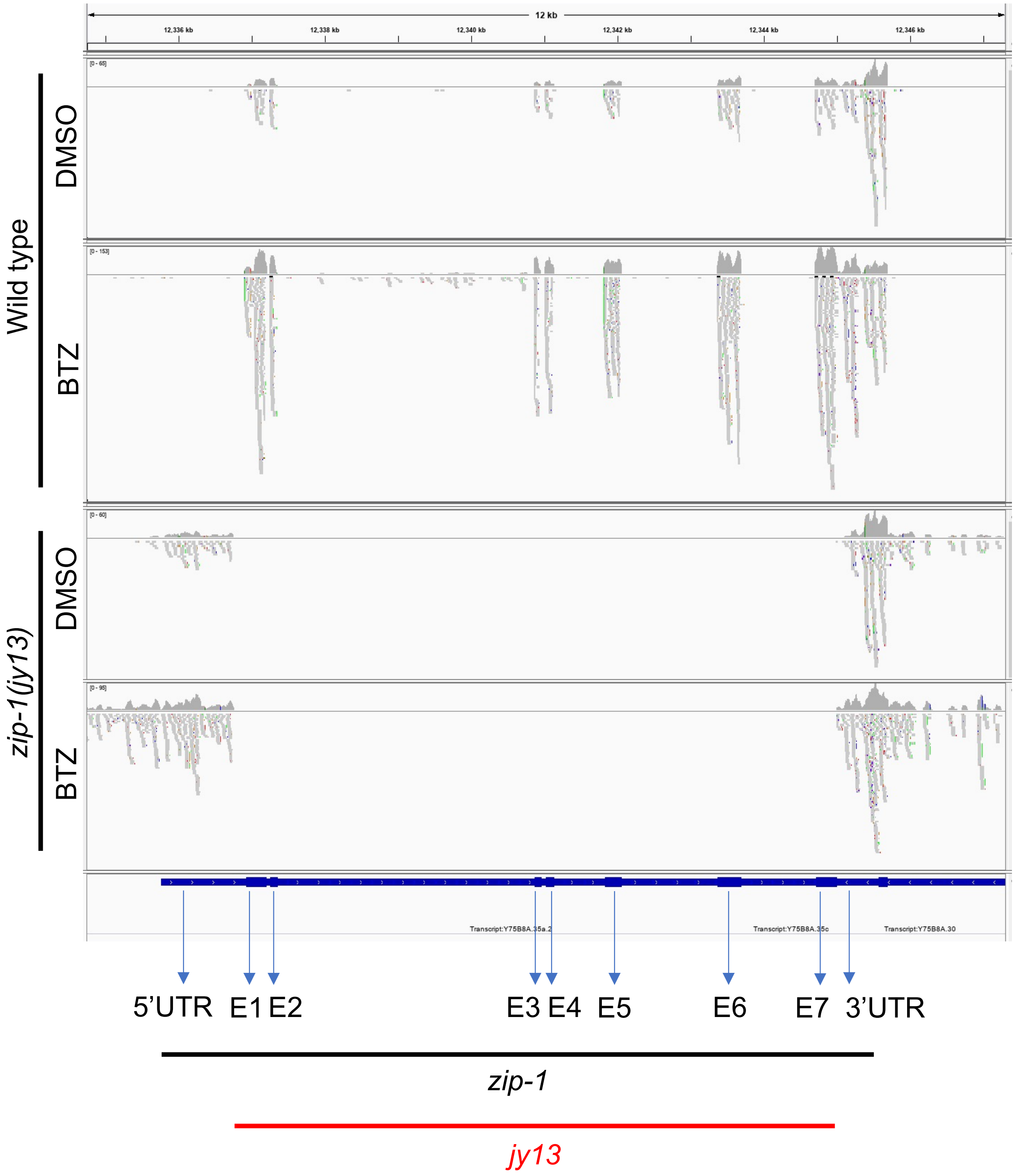

Supplementary Fig. 9: Alignment of mapped *zip-1* reads from RNA-seq analysis. Individual mapped reads and summary graphs are shown for DMSO and bortezomib treated wild-type N2 and *zip-1(jy13)* samples. Genomic location is indicated on the top of the graph. *zip-1* and *jy13* locations are indicated on the bottom. Exons of *zip-1* are labeled with E1-E7. Source data were uploaded to the NCBI GEO database with Accession number GSE183361.

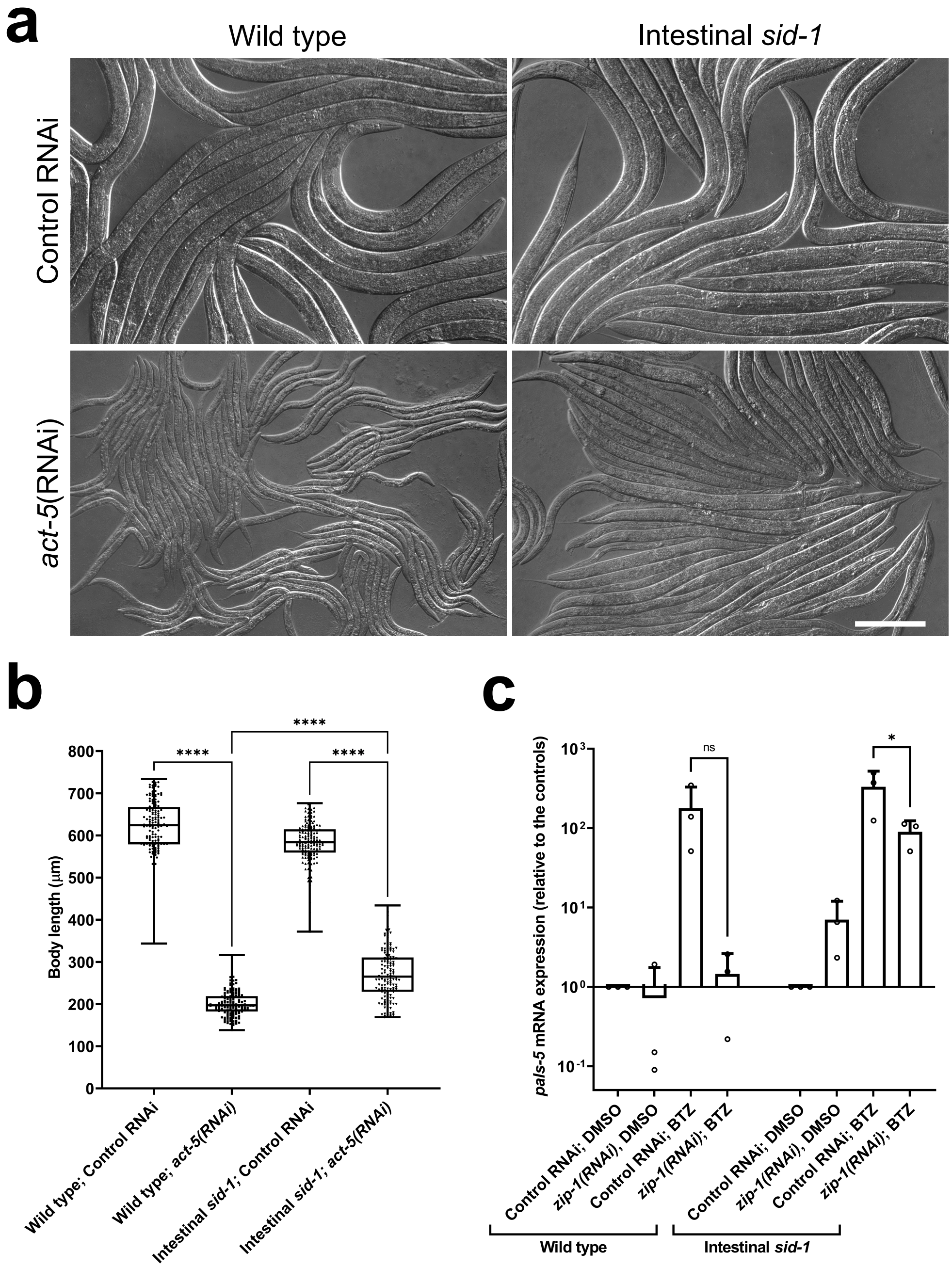

Supplementary Fig. 10: Intestine-specific *zip-1(RNAi)* (*sid-1* background) reduces *pals-5* mRNA induction. **a** Representative DIC images of wild-type and *sid-1* mutant animals following control and *act-5* RNAi treatments. Scale bar = 200 µm. **b** Graphical representation of body length measurements after 48 h incubation at 20°C. 150 animals were analyzed, 50 animals per each of three replicates. The box-and-whisker plots were used for data representation. The line in the box represents the median value, box bounds indicate the 25th and 75th percentiles, and whiskers extend from the box bounds to the minimum and maximum values. **c** qRT-PCR measurements of *pals-5* levels at the 30 min timepoint of bortezomib (BTZ) or DMSO treatments. The results are shown as fold change in gene expression relative to DMSO diluent control. Three independent experimental replicates were analyzed; the values for each replicate are indicated with circles.

Bar heights indicate mean values and error bars extend above. Error bars represent standard deviations. **b, c** A Kruskal-Wallis (**b**) and a one-tailed t-test (**c**) were used to calculate *p*-values; **** $p < 0.0001$; * $0.01 < p < 0.05$**;** ns indicates nonsignificant difference (p > 0.05). *p*-values in **b** for all comparisons $p < 0.0001$. *p*-values in **c** for WT control RNAi BTZ vs *zip-1(RNAi)* BTZ $p = 0.0559$; intestinal *sid-1* control RNAi BTZ vs *zip-1(RNAi)* BTZ $p = 0.0478$. Source data are provided as a Source Data file.

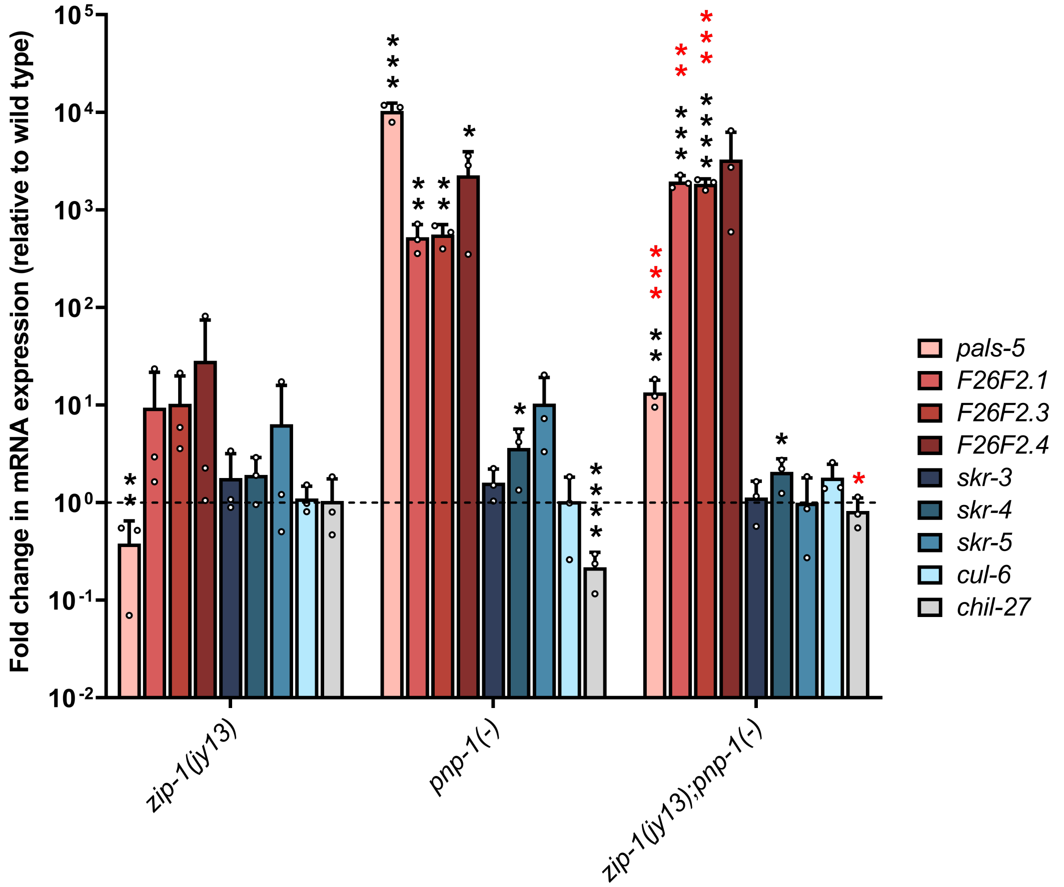

Supplementary Fig. 11: *zip-1* regulates expression of some IPR genes that are upregulated in *pnp-1(jy90)* mutants. qRT-PCR measurements of selected IPR genes and *chil-27* in wild-type, *zip-1(jy13)*, *pnp-1(-)* and *zip-1(jy13); pnp-1(-)* animals. The results are shown as the fold change in gene expression relative to control strain. All strains are in *jyIs8[pals-5p::gfp; myo-2p::mCherry]* strain background. Three independent experimental replicates were analyzed, the values for each replicate are indicated with circles. Bar heights indicate mean values and error bars extend above. Error bars represent standard deviations. A one-tailed t-test was used to calculate *p*-values; black asterisks represent significant difference between the labeled sample and the wild-type control; red asterisks represent significant difference between *zip-1(jy13); pnp-1(jy90)* and *pnp-1(jy90)*backgrounds; **** $p < 0.0001$; *** $p < 0.001$; ** $p < 0.01$; * $0.01 < p < 0.05$; *p*-values higher than 0.05 are not labeled. *p*-values for *zip-1(jy13)* vs WT: *pals-5* $p = 0.0081$; *pnp-1(jy90)* vs WT: *pals-5* $p = 0.0005$, *F26F2.1* $p = 0.0029$, *F26F2.3* $p = 0.0014$, *F26F2.4* $p = 0.0408$, *skr-4* $p = 0.0458$, *chil-27* $p < 0.0001$; *zip-1(jy13); pnp-1(jy90)* vs WT: *pals-5* $p = 0.0046$, *F26F2.1* $p = 0.0002$, *F26F2.3* $p < 0.0001$, *skr-4* $p = 0.0352$; *zip-1(jy13); pnp-1(jy90)* vs *pnp-1(jy90)*: *pals-5* $p = 0.0005$, *F26F2.1* $p = 0.0011$, *F26F2.3* $p = 0.0006$, *chil-27* $p = 0.0144$. All significant and non-significant *p*-values are provided as a Source Data file. Source data are provided as a Source Data file.

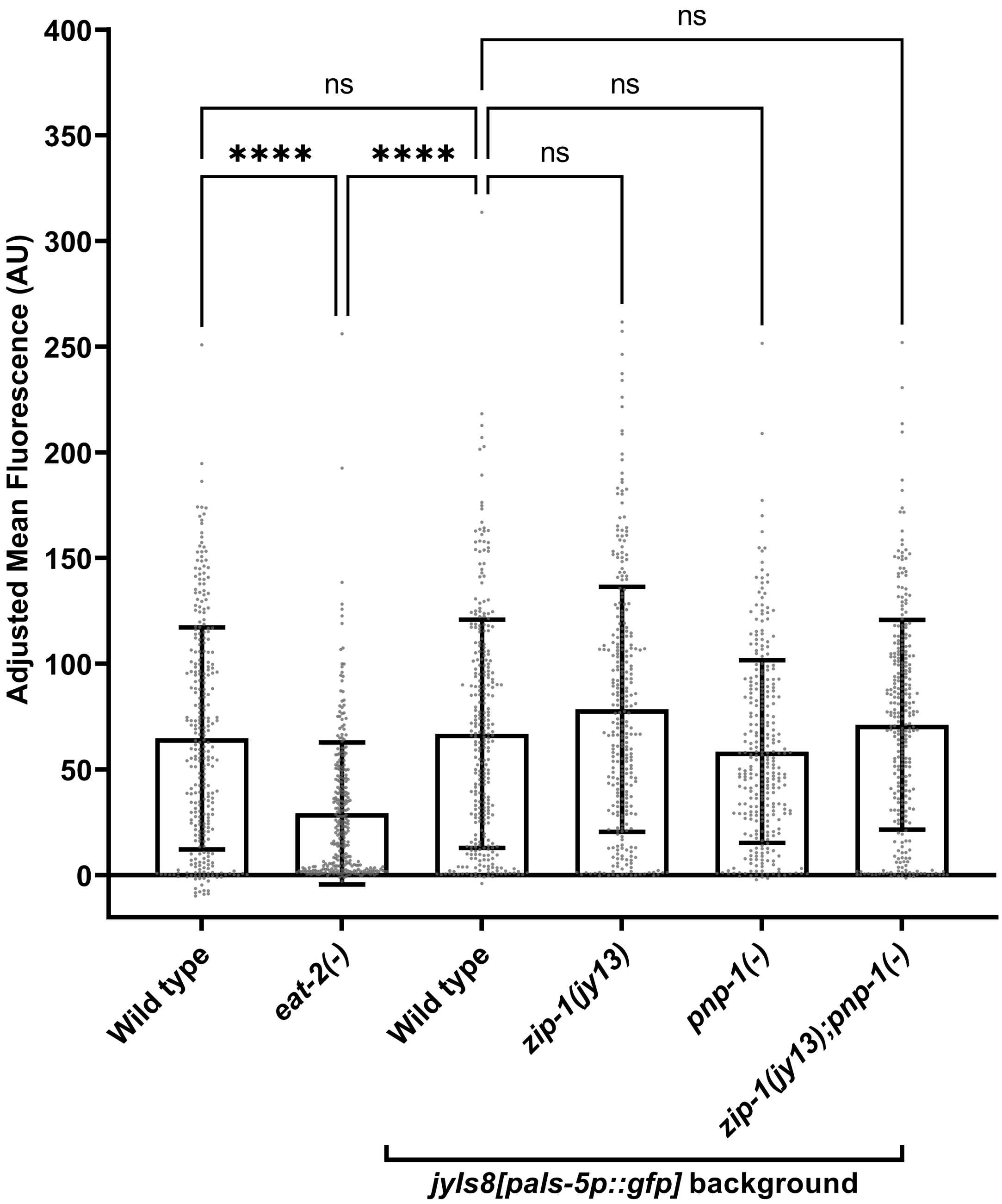

Supplementary Fig. 12: *zip-1(jy13)* and *pnp-1(jy90)* single and double mutants have similar accumulation of fluorescent beads. Quantification of fluorescent bead accumulation in the control strains (wild type and feeding-defective *eat-2* mutant), *zip-1(jy13); jyIs8*, *pnp-1(jy90); jyIs8* and *zip-1(jy13); pnp-1(jy90); jyIs8* mutants. Mean fluorescence was measured in 150 animals per genotype; background fluorescence was subtracted. Bar heights indicate mean values and error bars extend above and below. Error bars represent standard deviations. AU – arbitrary units. A Kruskal-Wallis test was used to calculate *p*-values; **** $p < 0.0001$; ns indicates nonsignificant difference ($p > 0.05$). *p*-values for WT vs *eat-2(ad465)* and *eat-2(ad465)* vs WT *jyIs8* $p < 0.0001$; WT vs WT *jyIs8*, WT *jyIs8* vs *pnp-1(jy90); jyIs8* and WT *jyIs8* vs *zip-1(jy13); pnp-1(jy90); jyIs8* $p > 0.9999$; WT vs *zip-1(jy13); jyIs8* $p = 0.2682$. Source data are provided as a Source Data file.

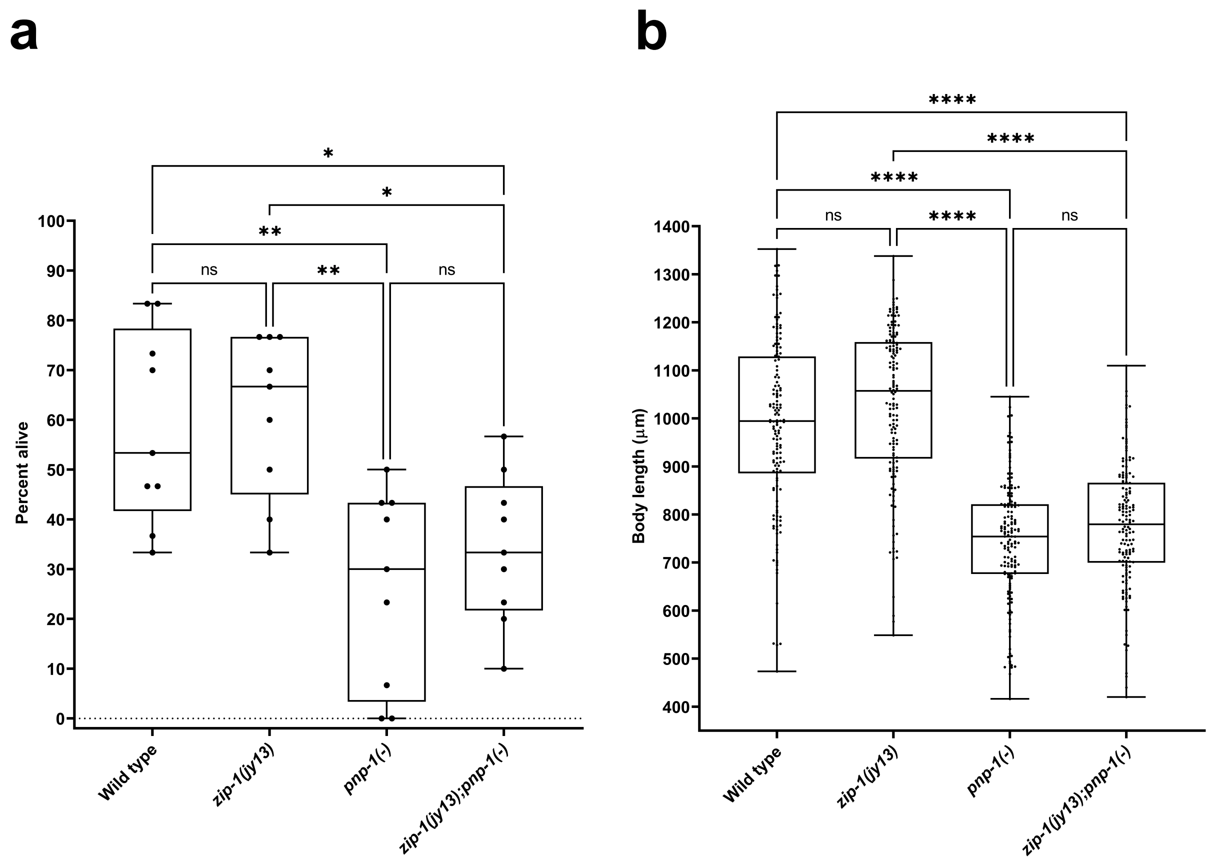

Supplementary Fig. 13: The phenotypes of increased sensitivity to heat shock and smaller size in *pnp-1(jy90)* mutants do not depend on *zip-1*. a Graphical representation of survival after heat shock. Nine biological replicates from three experiments are indicated with circles; 30 animals were analyzed in each replicate. **b** Graphical representation of body length measurements after 44 h incubation at 20°C. 150 animals were analyzed, 50 animals per each of three replicates. **a, b** The box-and-whisker plots were used for data representation. The line in the box represents the median value, box bounds indicate the 25th and 75th percentiles, and whiskers extend from the box bounds to the minimum and maximum values. All strains are in *jyIs8[pals-5p::gfp; myo-2p::mCherry]* strain background. A Kruskal-Wallis test was used to calculate *p*-values; **** $p < 0.0001$; ** $p < 0.01$; * $0.01 < p < 0.05$; ns indicates nonsignificant difference ($p > 0.05$). *p*-values in **a** for WT vs *zip-1(jy13)* $p = 0.9895$; WT vs *pnp-1(jy90)* $p = 0.0029$; WT vs *zip-1(jy13); pnp-1(jy90)* $p = 0.0308$; *zip-1(jy13)* vs *pnp-1(jy90)* $p = 0.0012$; *zip-1(jy13)* vs *zip-1(jy13); pnp-1(jy90)* $p = 0.0145$; *pnp-1(jy90)* vs *zip-1(jy13); pnp-1(jy90)* $p = 0.7897$. *p*-values in **b** for WT vs *zip-1(jy13)* $p = 0.4219$; WT vs *pnp-1(jy90)*, WT vs *zip-1(jy13); pnp-1(jy90)*, *zip-1(jy13)* vs *pnp-1(jy90)* and *zip-1(jy13)* vs *zip-1(jy13); pnp-1(jy90)* $p < 0.0001$; *pnp-1(jy90)* vs *zip-1(jy13); pnp-1(jy90)* $p = 0.6705$. Source data are provided as a Source Data file.

Supplementary Table 1: Wormcat analysis results. Overrepresented categories are listed for both analyzed time points. All catalog values represent number of genes in a specific category in the whole annotation list. p-values were determined using Bonferroni correction from the minimum hypergeometric scores calculated by the WormCat software. Bonferroni corrected *p*-values are shown in Fig. 4d.

**30 min bortezomib treatment**				
**Category**	**Number of genes**	**All catalog values**	***p*-value**	**Bonferroni corrected *p*-value**
**Stress response**	10	810	1.71029E-05	0.000222338
**4 h bortezomib treatment**				
**Category**	**Number of genes**	**All catalog values**	***p*-value**	**Bonferroni corrected *p*-value**
**mRNA functions**	51	390	3.08684E-17	9.8779E-16
**Transcription: general machinery**	23	145	3.8045E-10	1.21744E-08
**Nuclear pore**	13	36	5.62276E-10	1.79928E-08
**Signaling**	73	1193	1.29104E-08	4.13133E-07
**Development**	27	293	4.37601E-07	1.40032E-05
**Cytoskeleton**	30	369	1.19866E-06	3.83571E-05
**Proteolysis proteasome**	45	738	6.11019E-06	0.000195526
**DNA**	17	178	3.49017E-05	0.001116855

Supplementary Table 2: List of worm strains used in this study. Names of strains and their genotypes are listed.

Strain name	Genotype description
N2	wild type
DA465	*eat-2(ad465) II*
ERT054	*jyIs8[pals-5p::gfp, myo-2p::mCherry] X*
ERT071	*jyIs14[F26F2.1p::gfp, myo-2p::mCherry]*
ERT286	*pals-22(jy1) III; jyIs8[pals-5p::gfp, myo-2p::mCherry] X*
ERT29	*jyEx13[F26F2.1p::gfp, myo-2p::mCherry]*
ERT363	*unc-119(ed3) III; jyEx191[pals-5p::egfp::3xFLAG, unc-119(+)]*
ERT588	*zip-1(jy13) III; jyIs8[pals-5p::GFP, myo-2p::mCherry] X*
ERT590	*zip-1(jy13) III*
ERT592	*zip-1(jy14) III; jyIs8 [pals-5p::GFP, myo-2p::mCherry] X*
ERT705	*pnp-1(jy90) IV; jyIs8[pals-5p::GFP, myo-2p::mCherry] X*
ERT729	*jySi44 [pET689(pals-5p::NanoLuc::unc-54 3' UTR, unc-119 (+))] II; unc-119 (ed3) III*
ERT748	*zip-1(jy13) III; jyIs14[F26F2.1p::gfp, myo-2p::mCherry]*
ERT813	*zip-1(jy132[zip-1::gfp::sbp::3xFlag])*
ERT841	*zip-1(jy13) III; pnp-1(jy90) IV; jyIs8[pals-5p::gfp, myo-2p::mCherry] X*
ERT880	*pals-5(jy133) I*
ERT887	*jyIs44 [pET689 (pals-5p::NanoLuc::unc-54 3' UTR, unc-119 (+)] II; zip-1(jy13) unc-119 (ed3) III*
ERT1007	*zip-1(jy131[zip-1::gfp::sbp::3xFlag]) III; drh-1(jy110) IV*
MGH167	*sid-1(qt9) V; alxIs6[vha-6p::SID-1::SL2::GFP]*
NR222	*rde-1(ne219) V; kzIs9[pKK1260(lin-26p::NLS::gfp), pKK1253(lin-26p::rde-1), rol-6(su1006)]*
VP303	*rde-1(ne219) V; kbIs7[nhx-2p::rde-1; rol-6(su1006)]*

Supplementary Table 3: List of primers used in this study. Primer labels, descriptions and sequences are listed.

Primer number	Primer description	Primer sequence
1	*zip-1(jy13)* Forward Screening	5’-cgcgattctcgtagatcaaac-3’
2	*zip-1(jy14)* Forward Screening	5’-gcgaaaataggcgtggtattg-3’
3	*zip-1* Internal Forward Screening	5’-cttctggccttcctcattgat-3’
4	*zip-1* Reverse Screening	5’-ggagttcaaagtcgctgattg-3’
5	*gfp::sbp::3xFlag* Forward	5’-agcaatttgagccaagc-3’
6	*gfp::sbp::3xFlag* Reverse	5’-tttgtcatcgtcgtccttat-3’
7	*gfp::sbp::3xFlag* Forward with *zip-1* overhang	5’-gcgaatgcgattaaagtggtactcaatcaaatcacaagagaactgaaccgatacatggatggtgccaagcttgccgagcagcaggcttttgatgagaatttccagcaatttgagccaagc-3’
8	*gfp::sbp::3xFlag* Reverse with *zip-1* 3' UTR overhang	5’-gtgagaaattgagaaaaaaatggatttttgaagctttaaatataacatttggataaaaataactagttttcaaagatttttaatttttaatattttttcgctcaaaaaatccttcatttgtcatcgtcgtccttat-3’
9	*gfp*-specific Reverse Screening	5’-gtcttgtagttcccgtcatct-3’
10	*pals-5(jy133)* Forward Screening	5’- tgtagagggtcggaatatgc-3’
11	*pals-5(jy133)* Reverse Screening	5’-cagaatggacagtgtatcgc-3’
12	*snb-1* qRT-PCR Forward	5’-ccggataagaccatcttgacg-3’
13	*snb-1* qRT-PCR Reverse	5’-gacgacttcatcaacctgagc-3’
14	*pals-5* qRT-PCR Forward	5’-cattggaaagcgatattgga-3’
15	*pals-5* qRT-PCR Reverse	5’-tctccaggcacctatcttgtag-3’
16	*F26F2.1* qRT-PCR Forward	5’-tggaaccaggtcagagacac-3’
17	*F26F2.1* qRT-PCR Reverse	5’-ttgtgagaatttccgcgata-3’
18	*F26F2.3* qRT-PCR Forward	5’-ggaaagggaatgcattatgg-3’
19	*F26F2.3* qRT-PCR Reverse	5’-ccgcacggttatttctcat-3’
20	*F26F2.4* qRT-PCR Forward	5’-caacaatacactgcggatgg-3’
21	*F26F2.4* qRT-PCR Reverse	5’-tcgcactgttattcatctcca-3’
22	*skr-3* qRT-PCR Forward	5’-ccgacagccagaaacaaatca-3’
23	*skr-3* qRT-PCR Reverse	5’-tctgtgatggtcttggattgac-3’
24	*skr-4* qRT-PCR Forward	5’-ccgacagccagaaacaaatca-3’
25	*skr-4* qRT-PCR Reverse	5’-ggtcttggattggctgatcac-3’
26	*skr-5* qRT-PCR Forward	5’-cgaagagcaagatgtcaaaattg-3’
27	*skr-5* qRT-PCR Reverse	5’-agaagcttggattgattggca-3’
28	*cul-6* qRT-PCR Forward	5’-ctgggcttactcacaatgcc-3’
29	*cul-6* qRT-PCR Reverse	5’-gcagagttggcttgctgtaa-3’
30	*chil-27* qRT-PCR Forward	5'-tcaagtggaggactgcaaca-3'
31	*chil-27* qRT-PCR Reverse	5'-tgagttattttcggtagattccagt-3'
32	Orsay RNA1 qRT-PCR Forward	5'-acctcacaactgccatctaca-3'
33	Orsay RNA1 qRT-PCR Reverse	5′ -gacgcttccaagattggtattggt-3'

Supplementary Table 4: RNA-seq statistics. Numbers of total and mapped reads are given for each sample and each replicate. R1, R2 and R3 represent replicate 1, 2 and 3 respectively.

Sample name	Total reads	Mapped reads
N2 DMSO 0.5 h R1	24741912	22933141
N2 DMSO 0.5 h R2	25924996	23933650
N2 DMSO 0.5 h R3	26104291	24176026
N2 DMSO 4 h R1	27006387	24768399
N2 DMSO 4 h R2	26299275	24318612
N2 DMSO 4 h R3	27223331	25173138
N2 BTZ 0.5 h R1	25840460	23742750
N2 BTZ 0.5 h R2	28285206	26129222
N2 BTZ 0.5 h R3	26519896	24499694
N2 BTZ 4 h R1	25247160	23447221
N2 BTZ 4 h R2	25970252	24117470
N2 BTZ 4 h R3	25462110	23611688
zip-1(jy13) DMSO 0.5 h R1	25817283	24097889
zip-1(jy13) DMSO 0.5 h R2	26623992	24697010
zip-1(jy13) DMSO 0.5 h R3	25558331	23817145
zip-1(jy13) DMSO 4 h R1	24506002	22689324
zip-1(jy13) DMSO 4 h R2	28072318	26025881
zip-1(jy13) DMSO 4 h R3	26286843	24345490
zip-1(jy13) BTZ 0.5 h R1	27255831	25170595
zip-1(jy13) BTZ 0.5 h R2	26552739	24676577
zip-1(jy13) BTZ 0.5 h R3	28924046	26805076
zip-1(jy13) BTZ 4 h R1	25452767	23565691
zip-1(jy13) BTZ 4 h R2	25486693	23662044
zip-1(jy13) BTZ 4 h R3	26737835	24796481
