# Supplementary material for: The transcription factor ZIP-1 promotes resistance to intracellular infection in Caenorhabditis elegans
Source: Nat Commun. 2022 Jan 10;13:17. doi: 10.1038/s41467-021-27621-w (PMC8748929; doi:10.1038/s41467-021-27621-w)
Supplement: Supplementary file 2 — Description of Additional Supplementary Files [file 41467_2021_27621_MOESM2_ESM.pdf]

Description of Additional Supplementary Files

Supplementary Data 1: Results of RNAi screens

Supplementary Data 2: Differentially expressed genes in animals treated with bortezomib and DMSO

Supplementary Data 3: RNA-seq data comparisons

Supplementary Data 4: RNA-seq analysis, normalized counts
