# Supplementary material for: The transcription factor ZIP-1 promotes resistance to intracellular infection in Caenorhabditis elegans
Source: Nat Commun. 2022 Jan 10;13:17. doi: 10.1038/s41467-021-27621-w (PMC8748929; doi:10.1038/s41467-021-27621-w)
Supplement: Supplementary file 7 — Reporting summary [file 41467_2021_27621_MOESM7_ESM.pdf]

nature portfolio

Corresponding author(s): Emily R. Troemel

Last updated by author(s): 11/15/2021

Reporting Summary

Nature Portfolio wishes to improve the reproducibility of the work that we publish. This form provides structure for consistency and transparency in reporting. For further information on Nature Portfolio policies, see our Editorial Policies and the Editorial Policy Checklist.

Statistics

For all statistical analyses, confirm that the following items are present in the figure legend, table legend, main text, or Methods section.

n/a	Confirmed	
☐	☒	The exact sample size (*n*) for each experimental group/condition, given as a discrete number and unit of measurement
☐	☒	A statement on whether measurements were taken from distinct samples or whether the same sample was measured repeatedly
☐	☒	The statistical test(s) used AND whether they are one- or two-sided<br>*Only common tests should be described solely by name; describe more complex techniques in the Methods section.*
☐	☒	A description of all covariates tested
☒	☐	A description of any assumptions or corrections, such as tests of normality and adjustment for multiple comparisons
☐	☒	A full description of the statistical parameters including central tendency (e.g. means) or other basic estimates (e.g. regression coefficient) AND variation (e.g. standard deviation) or associated estimates of uncertainty (e.g. confidence intervals)
☐	☒	For null hypothesis testing, the test statistic (e.g. *F*, *t*, *r*) with confidence intervals, effect sizes, degrees of freedom and *P* value noted<br>*Give P values as exact values whenever suitable.*
☒	☐	For Bayesian analysis, information on the choice of priors and Markov chain Monte Carlo settings
☒	☐	For hierarchical and complex designs, identification of the appropriate level for tests and full reporting of outcomes
☒	☐	Estimates of effect sizes (e.g. Cohen's *d*, Pearson's *r*), indicating how they were calculated

Our web collection on statistics for biologists contains articles on many of the points above.

Software and code

Policy information about availability of computer code

Data collection	ZEN 2010 v 6.0.0.309 (Carl Zeiss), Axio Vison 40 v 4.8.2.0 (Carl Zeiss), MetaXpress 6 v 5.41.3 (Molecular Devices), Copas Biosort v 5.41.3 (Union Biometrica), Image Lab v 5.2.1 (BioRad), NOVOstar v 1.30 (BMG LABTECH), CFX Connect Real-Time PCR Detection System v. 3.1 (Bio-Rad).
Data analysis	Image Lab v 5.2.1 (BioRad), ImageJ 1.52p (FIJI) (Wayne Rasband NIH), Microsoft Office 16 Excel, GraphPad Prism 9, Mars Data Analysis Software v 2.00 (BMG LABTECH), StarSearch (http://rajlab.seas.upenn.edu/StarSearch/launch.html), R studio 1.3.1093, Galaxy (https://usegalaxy.org), WormCat 2.0 (http://wormcat.com).

For manuscripts utilizing custom algorithms or software that are central to the research but not yet described in published literature, software must be made available to editors and reviewers. We strongly encourage code deposition in a community repository (e.g. GitHub). See the Nature Portfolio guidelines for submitting code & software for further information.

Data

Policy information about availability of data

All manuscripts must include a data availability statement. This statement should provide the following information, where applicable:

- Accession codes, unique identifiers, or web links for publicly available datasets
- A description of any restrictions on data availability
- For clinical datasets or third party data, please ensure that the statement adheres to our policy

Data availability
The RNA-seq reads data generated in this study have been deposited in the NCBI GEO database under accession code GSE183361 [https://www.ncbi.nlm.nih.gov/geo/query/acc.cgi?acc=GSE183361]. Source data are provided with this paper. Supplementary Information file and Supplementary Data files are provided with this paper. C. elegans WS235 genome data used in RNA-seq analysis are publicly available (https://www.ncbi.nlm.nih.gov/assembly/GCF_000002985.6).

Field-specific reporting

Please select the one below that is the best fit for your research. If you are not sure, read the appropriate sections before making your selection.

☒ Life sciences ☐ Behavioural & social sciences ☐ Ecological, evolutionary & environmental sciences

For a reference copy of the document with all sections, see nature.com/documents/nr-reporting-summary-flat.pdf

Life sciences study design

All studies must disclose on these points even when the disclosure is negative.

Sample size	Sample sizes were not predetermined based on statistical methods. Sample sizes were chosen based on previously published methods that used the same types of assays, and provided statistically significant separation between strains of interest (e.g., Reddy, K. et al., Curr Biol, 2017; Reddy, K. et al., PLoS Pathog, 2019; Sowa J. et al., J Virol, 2020; Tecle E. et al., PLoS Pathog, 2021).
Data exclusions	sid-1 mutants showed poor IPR induction for reasons we do not understand. Therefore analysis of sid-1 mutants included only experimental replicates that had at least 50-fold increase in pals-5 expression levels on control RNAi plates following bortezomib treatment, as described in the methods section. This threshold allowed detection of a decrease in pals-5 induction in zip-1(RNAi) samples.
Replication	All experiments were performed in three experimental replicates except for:<br>- Fig. 1a: one or two experimental replicates, depending on RNAi clone<br>- Fig. 1c: two experimental replicates<br>- Fig. 6a: seven experimental replicates<br>- Fig. 6d: four experimental replicates<br>- Supplementary Figures 2, 3 and 6: two experimental replicates<br>All attempts of replication were successful.
Randomization	For all experiments animals were pooled and randomly allocated to different plates and submitted to different treatments.
Blinding	Samples were blindly allocated for RNAi screens. Samples were blinded for smFISH analysis. Automated analyses that rely on objective instrument measurements were not blinded (qRT-PCR, RNA-seq, fluorescence measurements).

Reporting for specific materials, systems and methods

We require information from authors about some types of materials, experimental systems and methods used in many studies. Here, indicate whether each material, system or method listed is relevant to your study. If you are not sure if a list item applies to your research, read the appropriate section before selecting a response.

Materials & experimental systems

n/a	Involved in the study	
☐	☒	Antibodies
☒	☐	Eukaryotic cell lines
☒	☐	Palaeontology and archaeology
☐	☒	Animals and other organisms
☒	☐	Human research participants
☒	☐	Clinical data
☒	☐	Dual use research of concern

Methods

n/a	Involved in the study	
☒	☐	ChIP-seq
☒	☐	Flow cytometry
☒	☐	MRI-based neuroimaging

Antibodies

Antibodies used	1. Tubulin primary antibody<br>Name: Monoclonal Anti-alpha-Tubulin antibody produced in mouse – clone DM1a, ascites fluid<br>Supplier: Sigma-Aldrich<br>Catalog #: T9026<br>Clone name: DM1A<br>Lot number: 047M4789V<br>2. PALS-5 primary antibody<br>Name: Polyclonal anti-PALS-5 antibody produced in Rabbit<br>Supplier: ProSci Inc. Antibody Services<br>Catalog #: n/a<br>Clone name: PAS-23740<br>Lot number: n/a

	3. Secondary antibody for Tubulin<br>Name: Goat Anti-Mouse IgG, H & L Chain Specific Peroxidase Conjugate<br>Supplier: Millipore<br>Catalog #: 401215<br>Clone name: n/a<br>Lot number: K3589978<br>4. Secondary antibody for PALS-5<br>Name: Goat Anti-Rabbit IgG, H & L Chain Specific Peroxidase Conjugate<br>Supplier: Millipore Sigma<br>Catalog #: 401315<br>Clone name: n/a<br>Lot number: 3611126
Validation	1. Tubulin primary antibody<br>Validation data: Independent antibody verification<br>https://www.sigmaaldrich.com/US/en/technical-documents/technical-article/protein-biology/immunohistochemistry/antibody-enhanced-validation#verification<br>Reference ID on RRID Portal: RRID:AB_477593<br>2. PALS-5 primary antibody<br>Validation data: ELISA (by Prosci), Independent antibody verification (two separately produced PALS-5 Abs both react), Genetic (reactivity enhanced in jy3 mutants and lost in pals-5 KO)<br>Reference ID on RRID Portal: AB_2893228<br>3. Secondary antibody for Tubulin<br>Reference ID on RRID Portal: AB_10682749<br>4. Secondary antibody for PALS-5<br>Reference ID on RRID Portal: AB_437787

Animals and other organisms

Policy information about studies involving animals; ARRIVE guidelines recommended for reporting animal research

Laboratory animals	Organism: Caenorhabditis elegans.<br>Sex: hermaphrodites.<br>Strains: names and genotypes are listed in Table S5.<br>Stage: experiments were performed using L4 stage animals, except for the infection assays (Fig. 2a and b, Fig. 6, Supplementary Fig. 3a and b and Supplementary Fig. 12) in which L1 stage animals were used.
Wild animals	Wild animals were not part of this study.
Field-collected samples	The study did not involve samples collected from the field.
Ethics oversight	No ethical approval is needed for laboratory use of C. elegans, because this is a nematode (invertebrate).

Note that full information on the approval of the study protocol must also be provided in the manuscript.
